# Supplementary material for: The genetic history of Mayotte and Madagascar cattle breeds mirrors the complex pattern of human exchanges in Western Indian Ocean
Source: G3 (Bethesda). 2022 Feb 7;12(4):jkac029. doi: 10.1093/g3journal/jkac029 (PMC8982424; doi:10.1093/g3journal/jkac029)
Supplement: jkac029_Supplemental_Figures [file jkac029_supplemental_figures.pdf]

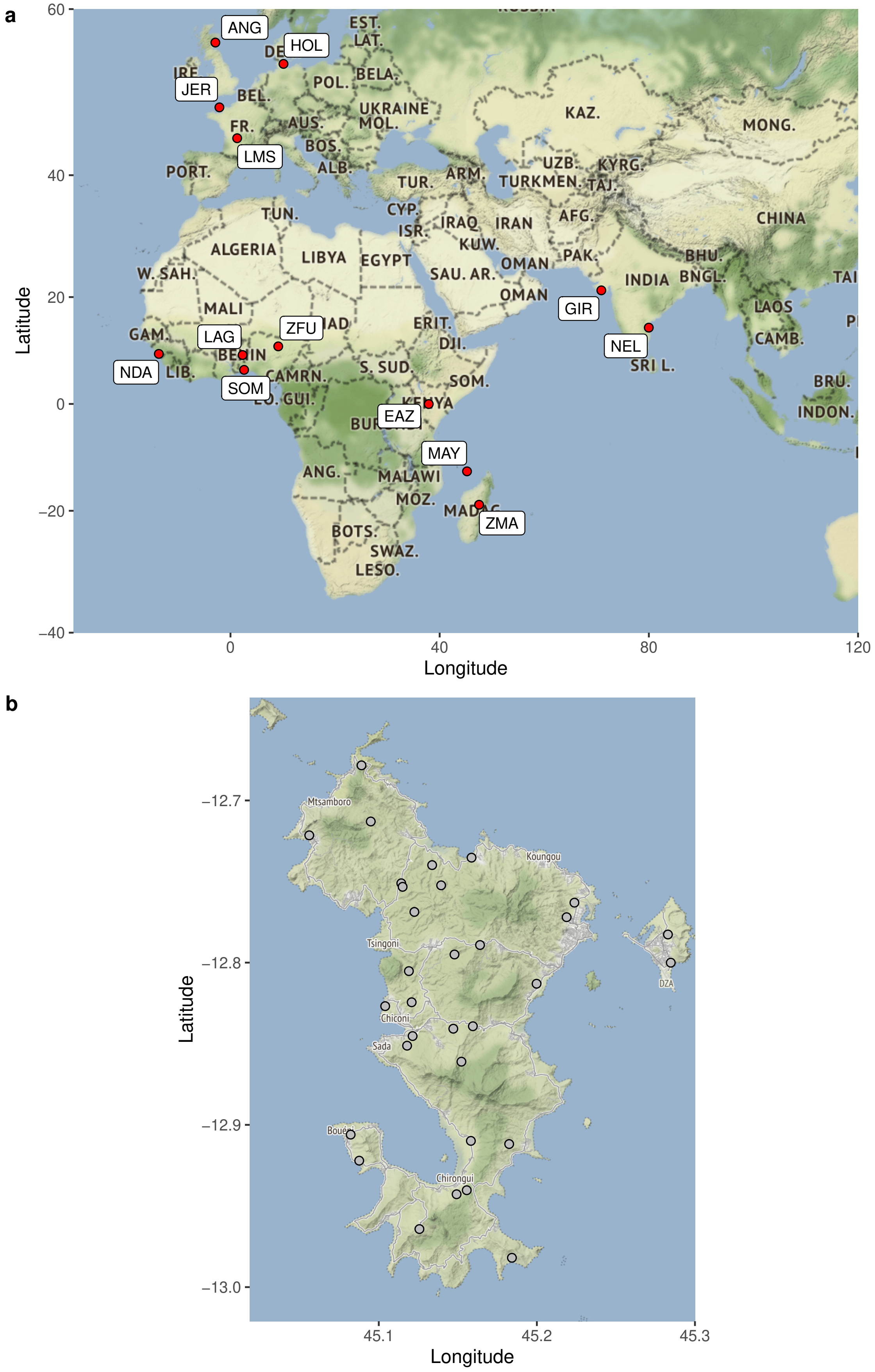

Figure S1 Maps and geographic coordinates of the populations studied (**a**) and the farms (**b**) where Zebus from Mayotte were sampled. Population coordinates correspond to the area of sampling except for those of Gir (GIR) and Nelore (NEL) populations which are located in their zone of origin.

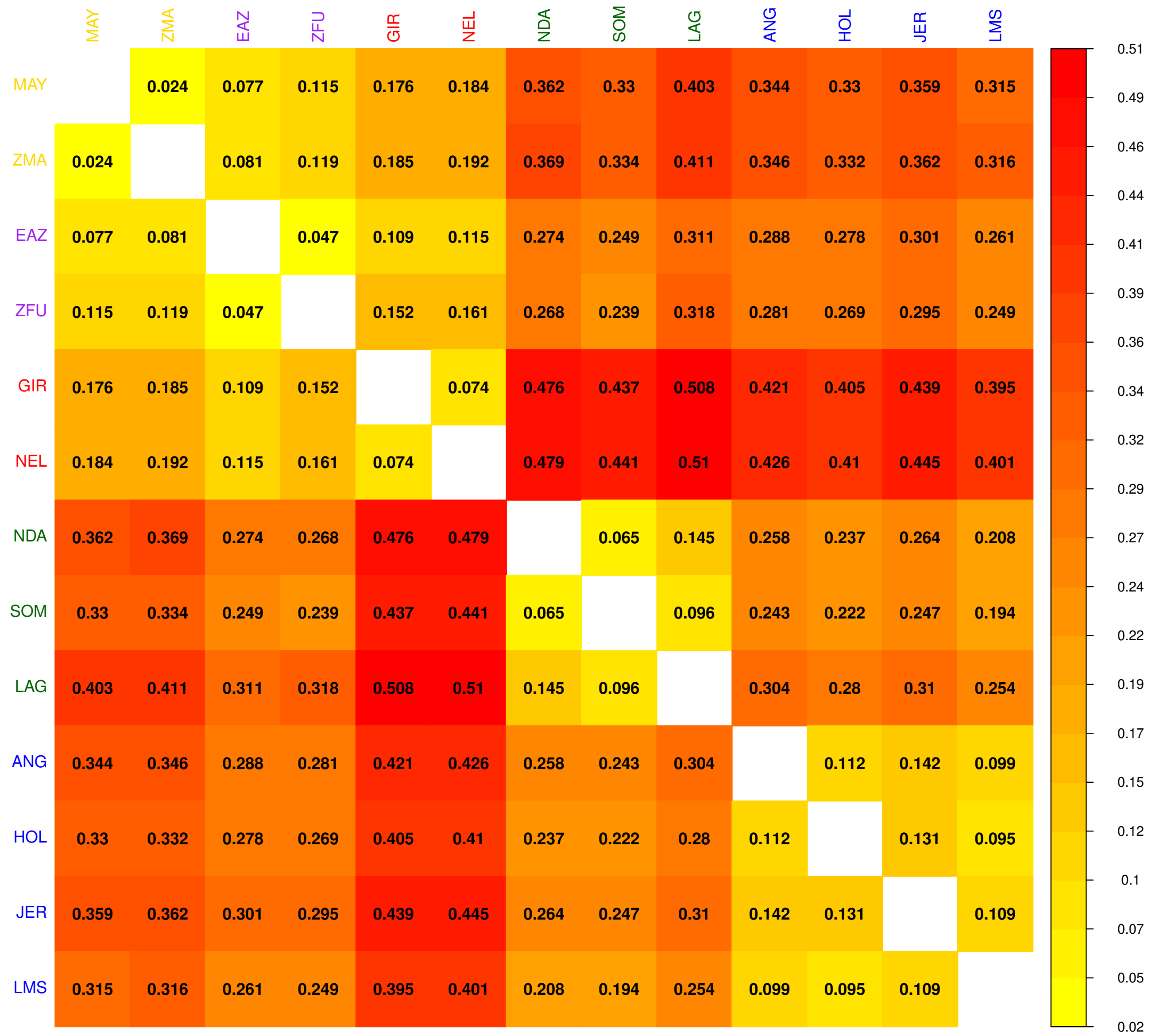

Figure S2 Pairwise-population F_{ST} among all the 13 populations.

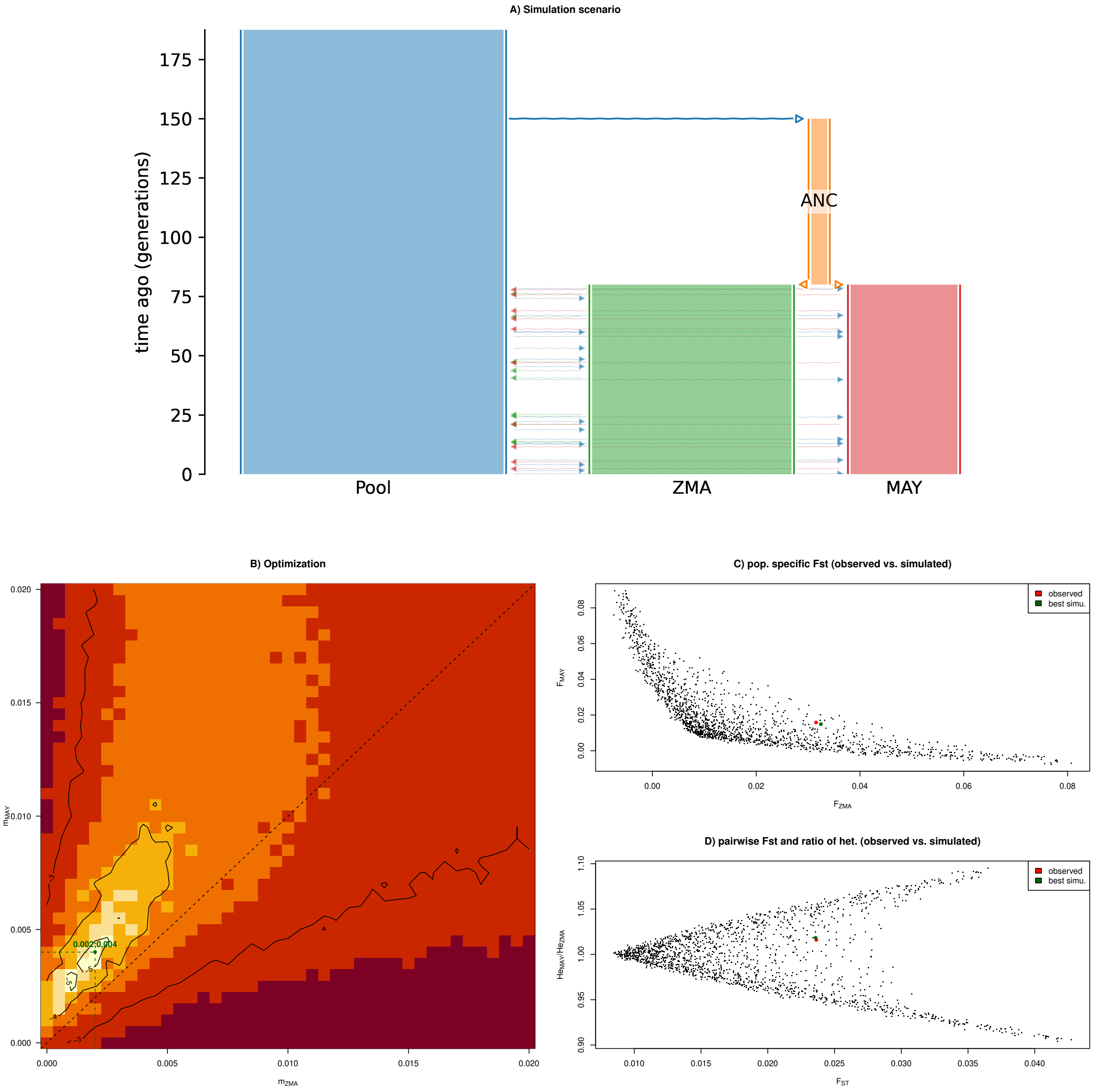

Figure S3 Simulation based evaluation of isolation models for ZMA and MAY with asymmetrical migration rates using `msprime` (Kelleher *et al.* 2016). A) Simulation scenario. Modeling of asymmetrical migration rate is performed via a (ghost) population ('Pool') with constant N_e =2500 that contribute to ZMA and MAY population with different symmetric migration rate (m_{ZMA} and m_{MAY} respectively) after the split from a common ancestral population named 'ANC' occurring $t = 80$ generations ago. The N_e for ZMA and MAY was set to 2,163 and 1,045 respectively which corresponded to the harmonic mean of the corresponding historical N_e estimates (Figure 3). Similarly the N_e for ANC was set to 250 and its divergence from the trunk population occurred $t = 150$ generations ago (i.e., the estimated timing of admixture for population ancestral to ZMA and MAY, named Ind. Oc. Zebus in Figure 2). B) Estimated distance δ (in a $\log_{10}$ scaled color gradient) between the observed data and data simulated over a grid of m_{zma} and m_{may} values (41 values ranging from 0 to 0.02 with a step of 0.005 for each parameter leading to a total of $1,681 = 41 \times 41$ simulated data sets). Each simulated data set consisted of 250 independent recombining segments of 5 Mb (assuming per-generation and per base mutation and recombination rates equal to $\mu = r = 10^{-8}$) for 23 ZMA and 30 MAY individuals and with a 4 generations sampling decay (as for the original data). The distance simulated and observed data was computed as $\delta = \left(F_{ST}^{obs} - F_{ST}^{sim}\right)^2 + \left(F_{zma}^{obs} - F_{zma}^{sim}\right)^2 + \left(F_{may}^{obs} - F_{may}^{sim}\right)^2 + \left(\rho_{He}^{obs} - \rho_{He}^{sim}\right)^2$ where F_{zma} (resp. F_{may} is the population-specific differentiation (e.g., $F_{zma} = \frac{Q_1^{zma} - Q_2^{zma,may}}{1 - Q_2^{zma,may}}$) computed from `poolfstat` estimates of Q_1 and Q_2 probability of identity (i.e., $F_{ST} = \frac{1}{2}\left(F_{zma} + F_{may}\right)$) and ρ_{He} is the ratio of within population MAY and ZMA heterozygosities ($= 1 - Q_1$). SNPs with a MAF< 0.01 (computed over MAY and ZMA combined samples) were discarded in both the observed and simulated to compute the statistics (leading to 518,315 SNPs for the observed data set and from 381,197 to 474,633 for the simulated ones). The optimal parameters values that minimized δ ($m_{zma} = 0.002$ and $m_{may} = 0.004$) are highlighted in green. C) Estimated F_{zma} and F_{may} for all the simulated data sets. The optimal parameters values (see B) and the values for the observed data set are highlighted in green and red respectively. D) Same as C) with estimated pairwise F_{ST} and ratio of heterozygosities ρ_{He}.

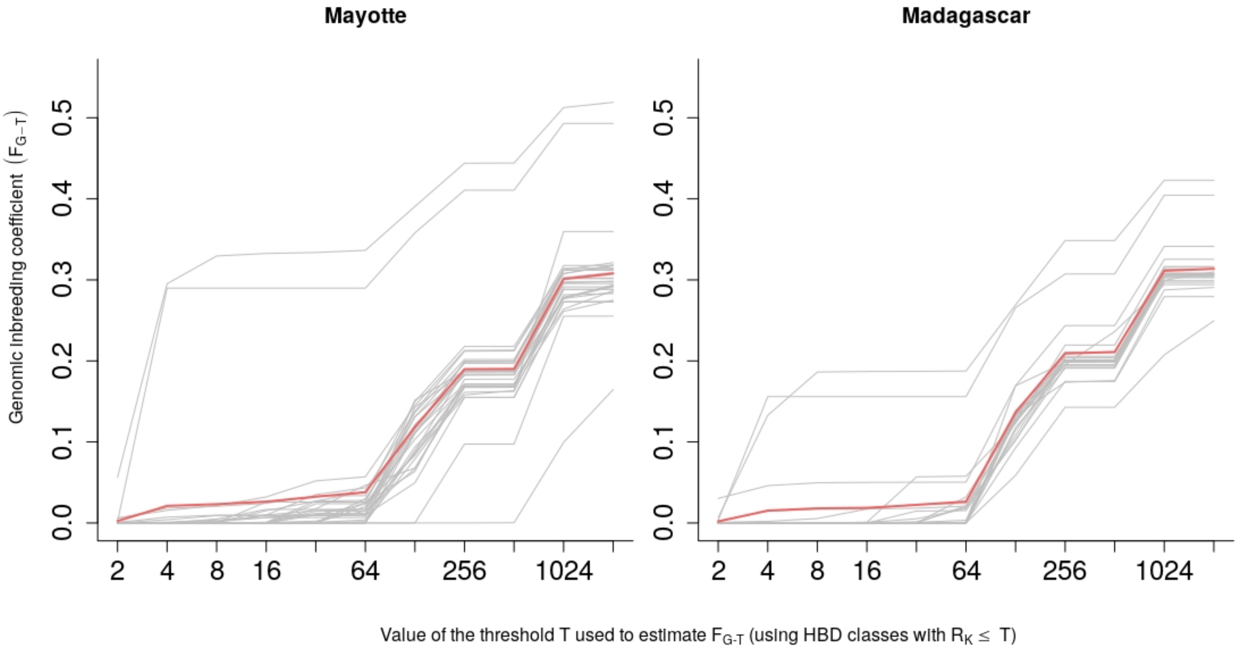

Figure S4 Proportion of the genome associated with different HBD classes at an individual level for Zebu from Mayotte (left) and Zebu from Madagascar (right). The figure was created with RzooRoh package (Bertrand *et al.* 2019)

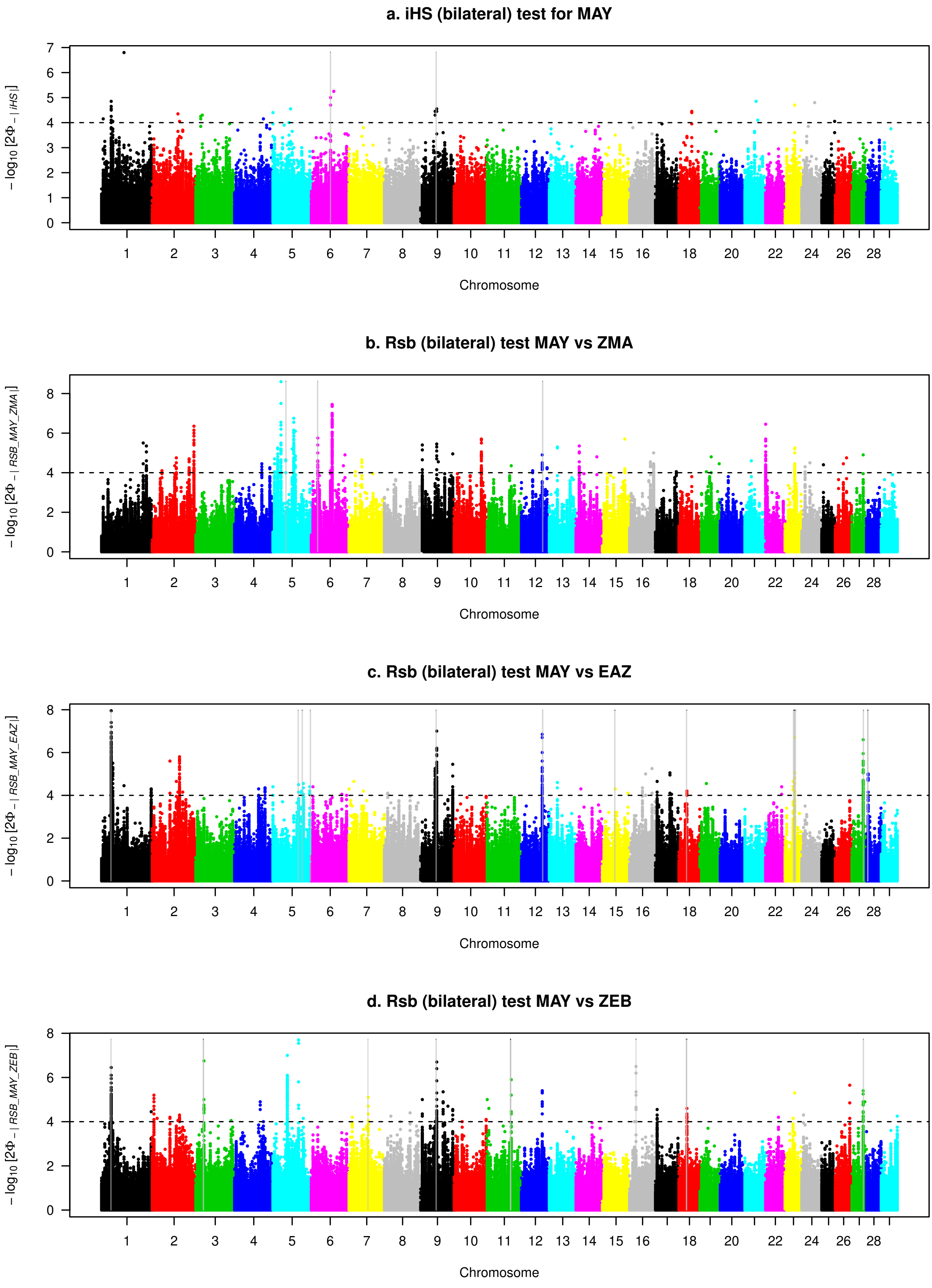

Figure S5 Manhattan plot of pvalues for iHS_{MAY} (a), $Rsb_{MAYvsZMA}$ (b), $Rsb_{MAYvsEAZ}$ (c) and $Rsb_{MAYvsZEB}$ (d) analyses. Positions of candidate genes are indicated by gray lines.

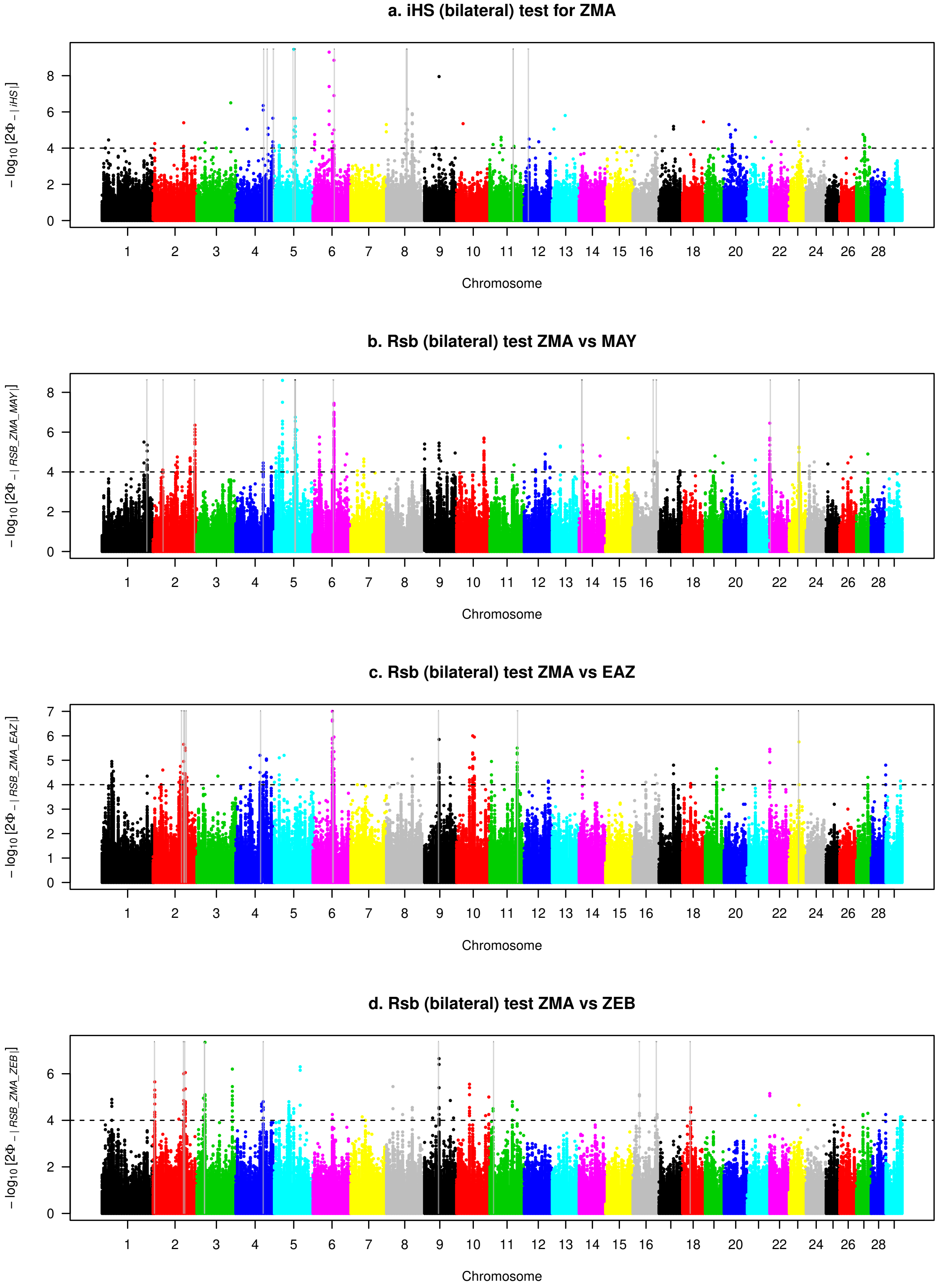

Figure S6 Manhattan plot of pvalues for iHS_{ZMA} (a), $Rsb_{ZMAvsMAY}$ (b), $Rsb_{ZMAvsEAZ}$ (c) and $Rsb_{ZMAvsZEB}$ (d) analyses. Positions of candidate genes are indicated by gray lines.

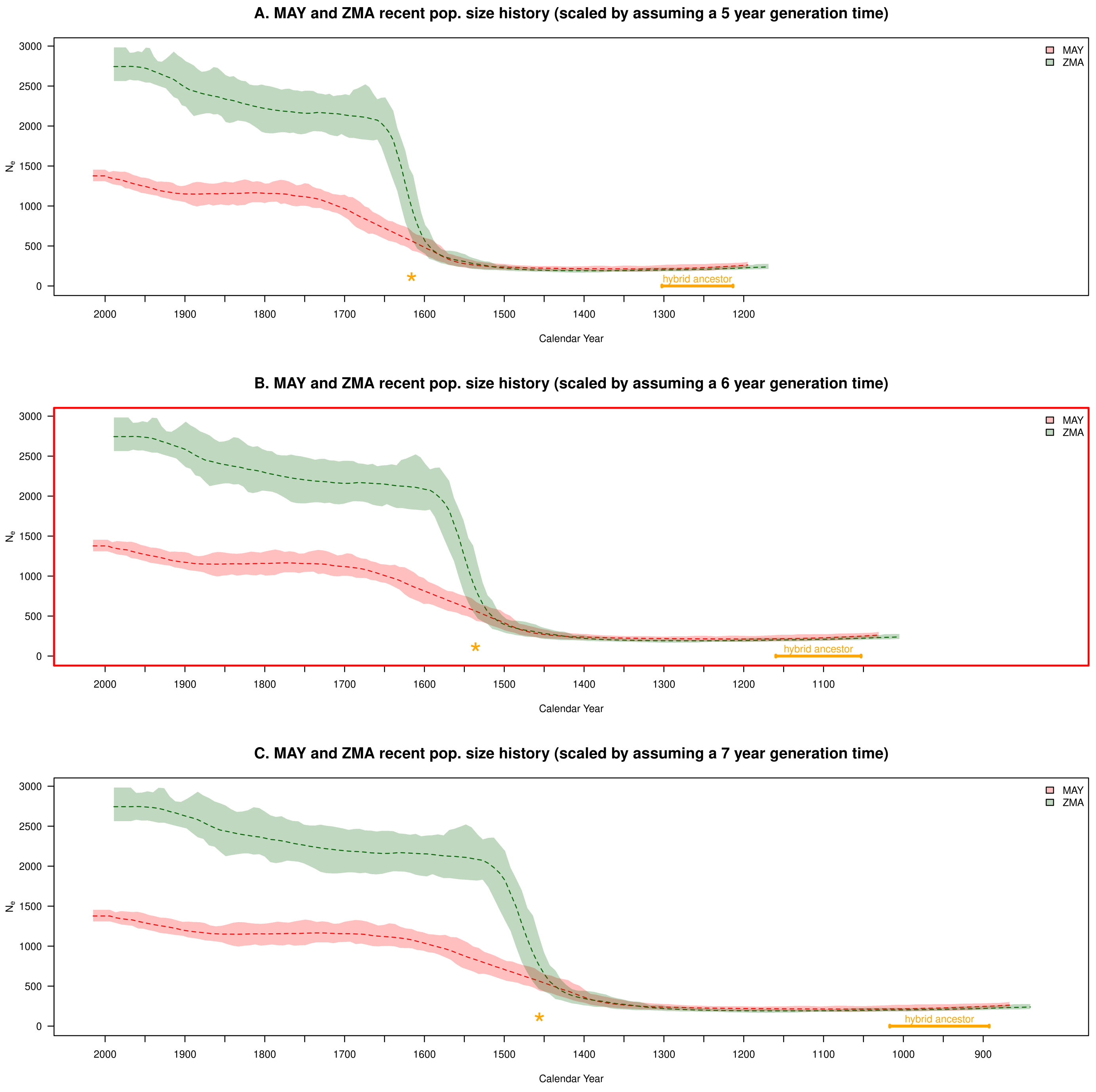

Figure S7 Comparison of population size histories (N_e) of MAY and ZMA populations (see Figure 3 of the main text) as a function of calendar years for three cattle generation times corresponding to A) five; B) six (same as Figure 3); or C) seven years. See Figure 3 legend for details.
